# Multiscale Cancer Modeling and In Silico Oncology: Emerging Computational Frontiers in Basic and Translational Cancer Research

**DOI:** 10.4172/2155-9538.1000e114

**Published:** 2013-05-25

**Authors:** Georgios S Stamatakos, Norbert Graf, Ravi Radhakrishnan

**Affiliations:** 1 In Silico Oncology Group, Institute of Communication and Computer Systems, National Technical University of Athens, Zografos, Greece; 2 Department of Pediatric Oncology and Hematology, University of Saarland, Homburg, Germany; 3 Department of Chemical and Biomolecular Engineering, Department of Bioengineering, Department of Biochemistry and Biophysics, University of Pennsylvania, Philadelphia, USA

Cancer cells contain numerous mutations in the genome that are present in most or all malignant cells of a tumor. While not all mutations are significant for cancer progression, a subset of them, often termed driver mutations, have presumably been selected because they confer a distinctive fitness advantage for malignant cells in a heterogeneous tumor microenvironment [1,2]. Correlative studies on clinical samples profiling such mutations in various cancer types suggest that such drivers confer fitness advantage by providing a gain of function in several categories of cancer cell signaling including cell adhesion and motility, signaling, transcriptional regulation, cellular metabolism, and intracellular trafficking [3,4]. One of the grand challenges of the understanding of cancer progression is to find mechanistic links between such alterations and the hall marks of cancers such as increased proliferation and survival, aggressive invasion and metastasis, evasion of cell death, and increased metabolism [5,6]. This challenge is also of quintessential clinical importance because patient outcome to therapy (both in terms of initial response to therapy and subsequent development of resistance to therapy) is now shown to depend on the genetic alterations (primary or acquired) in the individual patients [7,8]. Traditional methods in cell biology and cancer biology such as phospho-proteomics, immuno-precipitation, polymerase chain reaction, in-situ hybridization and molecular imaging, and direct sequencing, along with network-based theories and bioinformatics are reasonably poised to probe some of these altered traits, such as those connected with signaling, transcriptional regulation, and cellular metabolism, but are not directly amenable to dissect the underlying complexity of a cancer cell or a tumor tissue.

Two disciplines are beginning to emerge to address the void left in tackling these challenges: (1) multiscale cancer modeling on the basic cancer biology front addresses the need for building quantitative models at multiple length and time-scales in characterizing the underlying complexity in cancer signaling at the molecular, cellular, and tissue length-scales [9,10]; (2) in silico oncology on the translational front strives to develop computational platforms to guide the personalization of therapeutic regimens and the associated clinical decision process [11]. Many targets for therapeutic intervention/inhibition have been evaluated in the past few years on the basis of a strong promise provided by preclinical investigations. Nevertheless, experience has shown that the clinical trials are often unsuccessful when the drugs are administered to un-cohorted patient populations. Experience has accentuated the need to employ targeted therapies on select population of patients classified into cohorts based on molecular/genetic alterations [8]. Rapid genotyping platforms and advances in sequencing cancer genomes allow detection of genetic aberrations in clinical samples. This allows the identification of molecular targets in each individual patient, and also the tracking of acquired molecular changes (expression, mutation, etc.) during the progression of the disease or during treatment. Even with quantitative patient data involving protein expression (using immune histochemistry), gene copy number (fluorescence in-situ hybridization), mutations (DNA mutational analysis), and gene expression (using polymerase chain reaction or microarray technology), mapping the high-dimensional data to a set of viable cellular mechanisms and then infer treatment options is a daunting undertaking. A further problem is the heterogeneity of tumors that might show varied expression patterns in different areas of the tumor [12]. The question is, how precisely a tumor can be characterized by these techniques? That is, to relate the molecular profile of a given patient to disease prognosis and the efficacy of therapy is a grand challenge in clinical oncology. This represents the opportunity for in-silico modeling approaches.

## Multiscale Modeling of Molecular/Cellular Networks

Network analyses enable us to identify underlying patterns in a programmatic fashion and enable us to formally postulate functional relationships such as between gene expressions, transcription factor activation, as well as propose mechanisms of activation of cell signaling pathways [13]. A suite of network analysis and inference tools exists for the construction of network topologies (or interaction maps) surrounding transcriptional and proteomic measurements. This enables a bottom-up development of regulatory networks relevant to oncogenic signaling and provides a standardized platform to comprehend complex interactions/causalities, as well as generate hypothesis on functional maps relating the genomic, cell, and proteomic data. A summary of popular network analyses techniques is provided below.

### Inferring network topologies:

involves identification of interaction motifs such as feedback or feed forward loops, methods to infer bistability and robustness, construction and curation of Boolean networks and directed graphs using statistical inference techniques, information theory, and Bayesian analysis [13].

### Inferring gene and protein interaction networks:

involves applying dimensionality reduction techniques such as principal component analysis, hierarchical clustering and tree constructions, differential equation based kinetic modeling, rules-based modeling, stochastic and Monte Carlo based modeling of network dynamics [13].

### Multiscale modeling of functional interactions:

provides the means for constructing models of intra- as well as inter-cellular signaling networks with the ability to encode the resolution of molecular decisions at key nodes [9,14–16]. In particular, there is a need to analyze inter-cellular networks between cancer cells and immune cells of the same patient, which can prove to be significant for diagnosis etc. [17]. In the in-silico structural systems biology approach, network models of cellular signaling pathways (outlined above) [13] represent the highest level of modeling [15,16], where network analysis techniques are used to represent information flow in regulatory information cascades. Implementation of efficient approaches for parameter optimization and network sensitivity analysis are then utilized to analyze and contrast the transcriptional, proteomic and metabolic portraits of various cellular alterations/perturbations. Application of this approach to delineate the difference between wild type and oncogenically activated cell lines is described by Purvis et al. in [9]. The method has also been applied to predict the impacts of clinical mutational landscape on cellular activation mechanisms [14,18,11].

Beyond the modeling of intracellular signaling pathways, spatially regulated membrane-mediated phenomena such as cell adhesion and motility and intracellular trafficking are implicated in cancer progression. For example, altered endocytosis has been postulated as a mechanism by which malignant cells are able to generate hyper-proliferative signals (by modulating receptor tyrosine kinase activity), or decreased cell-adhesion and increased motility (by altering the trafficking of adhesion proteins or chemokine receptors) [19,20]. The recruitment of actin by chemokine receptor signaling has been correlated with metastatic behavior [21]. The formation of autophagosomes is correlated with large alterations in signaling and cellular metabolism in malignant cells [22]. Altered intracellular trafficking and subcellular localization in cancer and their effect on signaling can be studied using physically-based multiscale modeling methodologies by combining continuum field-based approaches with spatial and stochastic systems models for the signaling/trafficking microenvironment [23].

The ability to predict how small perturbations in molecular structure can lead to profoundly altered intracellular signaling pathways and subsequent cell-fate decisions is also crucial for predicting clinical outcome of cancer progression or efficacy of inhibition. In particular, the approaches summarized above enable the rationalization and prediction of the role and nature of molecular variability in networks by bridging the gap between molecular resolution/context, intracellular signaling, and pathway/target/biomarker validation personalized to the molecular profile/alterations in the individual patient. One viable mechanism for implementing these multiscale techniques to directly relate to the clinical setting is to create hyper-models, which are optimal and robust and can be used for predicting a physiological or a pathological function. Cell biology, histology, biomechanics, pharmacokinetics, pharmacodynamics, and radiobiology are some of the disciplines that are recruited in order to rationally link the molecular scale to the clinical one in the patient individualized context. Advanced mathematical, computational and visualization techniques play a crucial role in this endeavor. Such a hyper-model based decision support and treatment-planning system is the Oncosimulator [24–28]. The latter is at the same time a concept of multiscale integrative cancer biology, a complex algorithmic construct, a biomedical engineering system, and expectedly in the future, a clinical tool, which primarily aims at supporting the clinician in the process of optimizing cancer treatment in the patient individualized context through conducting experiments *in silico* i.e. on the computer. Additionally, it is a platform for simulating, investigating, better understanding and exploring the spatiotemporal *natural phenomenon* of cancer, facilitating the development of new treatment strategies, supporting the design and interpretation of clinico genomic trials and finally training doctors, researchers and interested patients alike. Imaging, histological, molecular, pharmacogenomic, clinical and treatment data at various time points constitute the main input of the Oncosimulator. A successful completion of its clinical adaptation, optimization and validation process currently under way is an obvious prerequisite for the clinical translation of the Oncosimulator.

The model predictions can be validated against multiscale clinical data in different cancer types. This vision brings to bear an in-silico platform, one that will allow clinicians and researchers alike to readily navigate within the heavily multidimensional space of multiscale data of each patient, to easily comprehend it, to run model simulations in silico, and finally to design treatment in the most scientifically grounded, quantitative and patient individualized fashion. This vision for in silico oncology represents a paradigm shift in medicine. The integrated computational and clinical considerations is also expected to improve interoperability of biomedical information and knowledge, increase the acceptance and use of realistic, clinically driven and oriented and validated models allowing researchers from different disciplines to exploit, share resources and develop new knowledge and improve accessibility to existing knowledge by bio-medical researchers. This need for multidisciplinary and integrated research is being fostered by several large-scale program projects such as the United States National Institutes of Health-funded Physical Sciences in Oncology Centers (PSOC), Integrative Cancer Biology Program (ICBP), Tumor Microenvironment Network (TMEN) [29, 30,31], and the European Commission Funded Projects such as Contra Cancrum, TUMOR, p-medicine, and Computational Horizons in Cancer (CHIC) [32,33,34].

The research leading to these results has received funding from the European Commission under the projects “ACGT: Advancing Clinico genomic Trials on Cancer” (FP6–2005-IST-026996), “Contra Cancrum: Clinically Oriented Translational Cancer Multilevel Modeling” (FP7-ICT-2007–2-223979), “TUMOR: Transatlantic Tumor Model Repositories” (FP7-ICT-2009.5.4–247754), “p-medicine: Personalized Medicine” (FP7-ICT-2009.5.3–270089, “DR THERAPAT: Digital radiation therapy patient” (FP7-ICT-2011.5.2–600852), “My Health Avatar: A Demonstration of 4D Digital Avatar Infrastructure for Access of Complete Patient Information” (FP7-ICT-2011.5.2–600929) and CHIC: Computational Horizons in Cancer: Computational Horizons In Cancer (CHIC): Developing Meta- and Hyper-Multiscale Models and Repositories for *In Silico* Oncology” (FP7-ICT-2011–9-600841). We also acknowledge funding for this work from US National Science Foundation Grants CBET-0853389 and CBET-0853539. Computational resources were available partly through US extreme science and engineering discovery environment (XSEDE) through a supercomputing resource allocation grant.

## References

[1] Loeb LA (2011) Human cancers express mutator phenotypes: origin, consequences and targeting. Nat Rev Cancer 11: 450–457. 21593786 10.1038/nrc3063 PMC4007007

[2] Haber DA, Settleman J (2007) Cancer: drivers and passengers. Nature 446: 145–146. 17344839 10.1038/446145a

[3] Sjoblom T, Jones S, Wood LD, Parsons DW, Lin J, (2006) The consensus coding sequences of human breast and colorectal cancers. Science 314: 268–274. 16959974 10.1126/science.1133427

[4] Greenman C, Stephens P, Smith R, Dalgliesh GL, Hunter C, (2007) Patterns of somatic mutation in human cancer genomes. Nature 446: 153–158. 17344846 10.1038/nature05610 PMC2712719

[5] Hanahan D, Weinberg RA (2011) Hallmarks of cancer: the next generation. Cell 144: 646–674. 21376230 10.1016/j.cell.2011.02.013

[6] Hanahan D, Weinberg RA (2000) The hallmarks of cancer. Cell 100: 57–70. 10647931 10.1016/s0092-8674(00)81683-9

[7] Rollins G (2011) The Era of Personalized Medicine in Cancer. Clinical Laboratory News, American Association of Clinical Chemistry 37.

[8] Normanno N, Rachiglio AM, Roma C, Fenizia F, Esposito C, (2013) Molecular diagnostics and personalized medicine in oncology: challenges and opportunities. J Cell Biochem 114: 514–524. 22991232 10.1002/jcb.24401

[9] Deisboeck TS, Stamatakos GS (2010) Multiscale Cancer Modeling of Cancer Mathematical and Computational Biology Series, Chapman & Hall-CRC, USA.

[10] Michor F, Liphardt J, Ferrari M, Widom J (2011) What does physics have to do with cancer? Nat Rev Cancer 11: 657–670. 21850037 10.1038/nrc3092 PMC3711102

[11] Proceedings of the 5th International Advanced Research Workshop on In Silico Oncology and Cancer Investigation, 2012 IEEE Xplore Print ISBN: 978-1-4673-5024-2 (Editor: G. S. Stamatakos). Open access available at http://www.5th-iarwisoci.iccs.ntua.gra and through the VPH Institute vph-institute.org/ (Editors: Stamatakos G and Dionysiou D) 10.1109/iarwisoci.2014.7034630 PMC8444280 34541587

[12] Heitzer E, Auer M, Hoffmann EM, Pichler M, Gasch C, (2013) Establishment of tumor-specific copy number alterations from plasma DNA of patients with cancer. Int J Cancer 133: 346–356. 23319339 10.1002/ijc.28030 PMC3708119

[13] Wang E (2010) Cancer Systems Biology CRC Press, Taylor and Francis, London, UK.

[14] Shih AJ, Telesco SE, Radhakrishnan R (2011) Analysis of Somatic Mutations in Cancer: Molecular Mechanisms of Activation in the ErbB Family of Receptor Tyrosine Kinases. Cancers (Basel) 3: 1195–1231. 21701703 10.3390/cancers3011195 PMC3119571

[15] Telesco SE, Shih AJ, Jia F, Radhakrishnan R (2011) A multiscale modeling approach to investigate molecular mechanisms of pseudokinase activation and drug resistance in the HER3/ErbB3 receptor tyrosine kinase signaling network. Mol Biosyst 7: 2066–2080. 21509365 10.1039/c0mb00345j PMC3138520

[16] Telesco SE, Radhakrishnan R (2012) Structural systems biology and multiscale signaling models. Ann Biomed Eng 40: 2295–2306. 22539148 10.1007/s10439-012-0576-6 PMC4612526

[17] Keller A, Leidinger P, Bauer A, Elsharawy A, Haas J, (2011) Toward the blood-borne miRNome of human diseases. Nat Methods 8: 841–843. 21892151 10.1038/nmeth.1682

[18] Bresler SC, Wood AC, Haglund EA, Courtright J, Belcastro LT, (2011) Differential inhibitor sensitivity of anaplastic lymphoma kinase variants found in neuroblastoma. Sci Transl Med 3: 108ra114. 10.1126/scitranslmed.3002950 PMC3319004 22072639

[19] Mosesson Y, Mills GB, Yarden Y (2008) Derailed endocytosis: an emerging feature of cancer. Nat Rev Cancer 8: 835–850. 18948996 10.1038/nrc2521

[20] Aust DE, Terdiman JP, Willenbucher RF, Chew K, Ferrell L, (2001) Altered distribution of beta-catenin, and its binding proteins E-cadherin and APC, in ulcerative colitis-related colorectal cancers. Mod Pathol 14: 29–39. 11211307 10.1038/modpathol.3880253

[21] Müller A, Homey B, Soto H, Ge N, Catron D, (2001) Involvement of chemokine receptors in breast cancer metastasis. Nature 410: 50–56. 11242036 10.1038/35065016

[22] Tooze SA, Jefferies HB, Kalie E, Longatti A, McAlpine FE, (2011) Trafficking and signaling in mammalian autophagy. IUBMB Life 62: 503–512. 10.1002/iub.334 20552641

[23] Ramanan V, Agrawal NJ, Liu J, Engles S, Toy R, (2011) Systems biology and physical biology of clathrin-mediated endocytosis. Integr Biol (Camb) 3: 803–815. 21792431 10.1039/c1ib00036e PMC3153420

[24] Stamatakos GS (2011) In Silico Oncology Part I: Clinically Oriented Cancer Multilevel Modeling Based on Discrete Event Simulation. In: Deisboeck T, Stamatakos G(Eds), CRC Press, Boca Raton, Florida, USA.

[25] Stamatakos GS, Georgiadi EC, Graf N, Kolokotroni EA, Dionysiou DD (2011) Exploiting clinical trial data drastically narrows the window of possible solutions to the problem of clinical adaptation of a multiscale cancer model. PLoS One 6: e17594. 21407827 10.1371/journal.pone.0017594 PMC3048172

[26] Graf N, Hoppe A, Georgiadi E, Belleman R, Desmedt C, (2009) ‘In silico’ oncology for clinical decision making in the context of nephroblastoma. Klin Padiatr 221: 141–149. 19437361 10.1055/s-0029-1216368

[27] Dionysiou DD, Stamatakos GS, Uzunoglu NK, Nikita KS, Marioli A (2004) A four-dimensional simulation model of tumour response to radiotherapy in vivo: parametric validation considering radiosensitivity, genetic profile and fractionation. J Theor Biol 230: 1–20. 15275995 10.1016/j.jtbi.2004.03.024

[28] Stamatakos GS, Dionysiou DD, Zacharaki EI, Mouravliansky NA, Nikita KS (2002) In silico radiation oncology: combining novel simulation algorithms with current visualization techniques. Proceedings of the IEEE: Special Issue on Bioinformatics: Advances and Challenges. 90: 1764–1777.

[29] Physical Sciences in Oncology Centers: http://physics.cancer.gov

[30] Integrative Cancer Biology Program: http://icbp.nci.nih.gov

[31] Tumor Microenvironment Network: http://tmen.nci.nih.gov

[32] ContraCancrum: http://www.contracancrum.eu

[33] TUMOR: http://www.tumor-project.eu

[34] P-Medicine: http://www.p-medicine.eu, and Computational Horizons in Cancer: http://www.chic-vph.eu/

